# Prognostic marker Musashi-2 modulates DNA damage response and radioresistance in diffuse large B-cell lymphoma

**DOI:** 10.3389/fcell.2025.1575483

**Published:** 2025-08-06

**Authors:** Timo Habig, Lasse Reichstein, Kathrin A. Brücksken, Mark Sicking, Jan Labisch, Michael Oertel, Eberhard Korsching, Georg Lenz, Stephan Hailfinger, Burkhard Greve, Fabian M. Troschel, Hans Theodor Eich

**Affiliations:** ^1^ Department of Radiation Oncology, University Hospital Münster, Münster, Germany; ^2^ Department of Medicine A, University Hospital Münster, Münster, Germany; ^3^ Cancer and Complex Systems Research Group, Medical Faculty, Münster University, Münster, Germany

**Keywords:** Musashi-2 (MSI2), DLBCL-diffuse large B-cell lymphoma, DNA damage (DDR), radiotherapy, NOTCH signaling pathway

## Abstract

**Introduction:**

Treatment resistance is a major hurdle in diffuse large B-cell lymphoma (DLBCL) therapy. Here, we assessed the relevance of the Musashi (MSI) RNA-binding protein family for DLBCL treatment efficacy. As important gene expression regulators, these proteins have previously been associated with tumorigenesis, treatment failure, and reduced survival in other malignancies, making them promising candidates for assessment in the context of DLBCL outcome and therapy resistance.

**Methods:**

We first leveraged publicly available gene expression studies to determine expression and prognostic relevance of MSI1 and MSI2 in DLBCL. We then characterized MSI2 co-expressed therapy-relevant signaling. After performing MSI2 knockdown experiments we investigated subsequent effects on DLBCL gene expression *in vitro* using qPCR, Western blot, protein arrays, and flow cytometry. Finally, cell viability assays and clonogenic assessments were used to assess resistance to radiation, vincristine, and doxorubicin chemotherapy.

**Results:**

MSI2 was overexpressed and prognostically unfavorable in univariable and multivariable analyses in DLBCL while MSI1 showed very low expression. High MSI2 expression was associated with increased stemness and DNA repair signaling. MSI2 knockdown led to a loss of stemness-associated markers and compromised DNA repair protein activation while increasing radiation-induced DNA double-strand break levels. Cell survival after either radiotherapy, vincristine or doxorubicin chemotherapy was impaired after MSI2 knockdown in follow-up analyses, suggesting a radio- and chemosensitizing effect.

**Discussion:**

We propose that MSI2, a prognostic marker, may modulate the susceptibility of DLBCL towards genotoxic therapy. Suppressing MSI2 may hold promise to sensitize DLBCL to DNA-targeted treatment.

## 1 Introduction

Diffuse large B-cell lymphoma (DLBCL) is a heterogeneous group of aggressive B cell-derived lymphomas, subdivided into the activated B-cell type (ABC), the germinal center B-cell type (GCB) and some minor, partially genetically defined groups (Lenz et al., 2008b; Sehn and Salles, 2021). The ABC subtype is characterized by a comparatively higher therapy resistance and reduced outcomes (Chapuy et al., 2018; Sehn and Salles, 2021). First-line standard of care for DLBCL includes R-CHOP chemo-immunotherapy with response-adapted, positron emission tomography (PET)-guided adjuvant radiotherapy (Levis and Oertel, 2025). Despite sophisticated therapy, roughly 40% of patients do not achieve cure (He and Kridel, 2021), highlighting the need to better understand mechanisms of therapy resistance (Sehn and Salles, 2021; Zhang et al., 2023).

The Musashi RNA-binding protein family, including Musashi-1 (MSI1) and Musashi-2 (MSI2), is highly conserved and strongly involved in neurogenesis (Kudinov et al., 2017). Musashi-1 was first described in neural cells of *Drosophila melanogaster* (Nakamura et al., 1994), with Musashi-2 later identified. Both proteins show an estimated amino acid overlap of 75% (Kudinov et al., 2017). They are known as important translational regulators by binding to untranslated regions (UTR), especially the 3’UTR, of specific mRNAs using RNA recognition motifs. Upon binding, they may initiate or repress translation of mRNAs, thus shaping cellular gene expression. Consequently, the Musashi RNA-binding proteins have been described as key regulators of cellular function (Battelli et al., 2006; Karmakar et al., 2022). Physiologically, MSI protein expression has been closely linked to stemness and tissue regeneration. However, the MSI proteins have also been found to be dysregulated in multiple solid malignancies (Jiang et al., 2022). There, they have been linked to therapy resistance via their association with cancer stem cells (CSCs) (Fox et al., 2015; Nguyen et al., 2020; Löblein et al., 2021; Topchu et al., 2021; Yiming et al., 2021). CSCs are a subgroup of highly therapy resistant tumor cells, oftentimes found in specific extracellular milieus and characterized by reduced cell cycle activity (Reya et al., 2001). Evidence suggests that therapy resistance may also be associated with CSC-associated stemness gene expression in hematologic malignancies, including in Hodgkin’s Lymphoma (Troschel et al., 2021a), or T cell leukemia (Ellisen et al., 1991; Reya et al., 2001). An association between CSC-associated stemness genes and DLBCL has also been proposed (Kharas and Lengner, 2017; Ryu et al., 2017; Chang et al., 2020). In this setting, Musashi protein family member MSI2 has recently been linked to tumorigenicity and stemness in aggressive mantle cell lymphoma (Sureda-Gómez et al., 2023) as well as to resistance to PRMT5-targeted therapy in B-cell lymphoma (Erazo et al., 2022), indicating a potential relevance for therapy of hematological malignancies. In addition to cancer stem cells, recent studies in lung cancer, glioblastoma and triple-negative breast cancer have demonstrated a direct link between MSI proteins expression and the DNA damage response as a contributor to therapy resistance. MSI proteins were shown to play a critical role by modulating DNA damage response pathways, resulting in a diminished capacity of MSI-deficient cells to repair DNA damage (Lin et al., 2018; Qu et al., 2024; Barber et al., 2025; Brücksken et al., 2025). Other important pathways linked to MSI proteins in multiple malignancies include proliferation, cell cycle, migration, and invasion (Liu et al., 2014; Yang et al., 2016).

**FIGURE 1:**
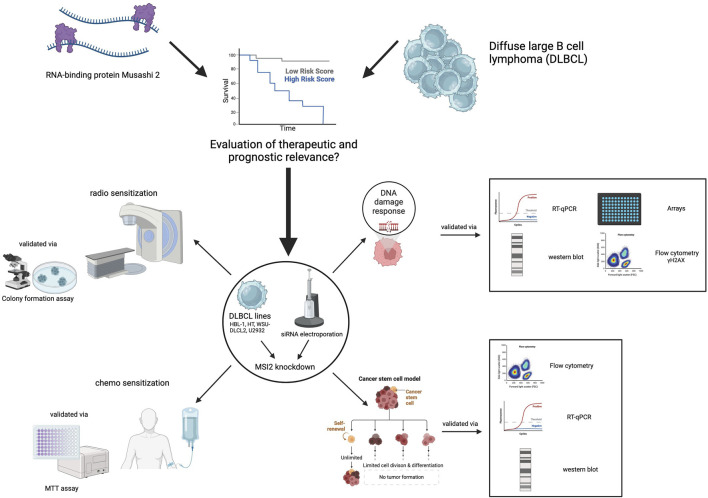
Graphical abstract. Treatment resistance is a major challenge in DLBCL therapy. We investigated the role of the Musashi (MSI) RNA-binding protein family for treatment efficacy, focusing on MSI2. Public gene expression data showed that MSI2 is overexpressed and associated with poor prognosis, while MSI1 is minimally expressed. MSI2 knockdown reduced stemness markers, compromised DNA repair, and increased radiation-induced DNA double-strand breaks. Follow-up experiments revealed that MSI2 knockdown sensitized DLBCL cells to radiotherapy, vincristine and doxorubicin chemotherapy. These findings suggest that targeting MSI2 could improve DLBCL response to genotoxic therapies. Created with BioRender.com.

In the present study, we aimed to assess the expression and prognostic significance of the MSI family in DLBCL as well as to investigate its relevance for first-line DLBCL therapy resistance. An overview of this study as a graphical abstract is presented in Figure 1.

## 2 Methods

### 2.1 Database analysis

We obtained normalized gene expression data from the datasets GSE32018 (Gómez-Abad et al., 2011), GSE50721 (Hardee et al., 2013), and GSE181063 (Painter et al., 2019; Lacy et al., 2020) and performed targeted analyses for *MSI1* and *MSI2* expression. For overall survival (OS) analyses, normalized *MSI2* expression and outcome data as well as DLBCL subtyping and International Prognostic Index (IPI) information were abstracted from the GSE181063 (Painter et al., 2019; Lacy et al., 2020), GSE31312 (Visco et al., 2012), GSE10846 (Lenz et al., 2008a), and GSE87371 (Dubois et al., 2017) datasets. Using the largest available cohort, GSE181063, we first dichotomized *MSI2* expression by median for a high-expressing and a low-expressing group. We then generated Kaplan Meier plots and performed log-rank tests on these groups to assess univariable associations of *MSI2* with OS. We also assessed whether patient characteristics showed associations with *MSI2* expression using Mann Whitney U tests or Spearman’s correlation, as appropriate. Then, using all four datasets for validation, we performed multivariable time-to-event cox proportional hazard regressions for OS independently for each dataset, including the highest-expressed *MSI2* read from the sequencing data as well as two established prognostic factors, DLBCL subtyping and IPI scoring. Here, hazard ratio (HR), confidence interval (CI), and *p* value are presented.

To determine mRNAs co-expressed with *MSI2* we leveraged the “The Cancer Genome Atlas” (TCGA) database on Lymphoid Neoplasm Diffuse Large B-cell Lymphoma (TCGA-DLBC), as analyzed by the The University of ALabama at Birmingham CANcer data analysis Portal (UALCAN) (Chandrashekar et al., 2017; 2022). This resulting list of *MSI2* co-expressed mRNAs was then submitted to the Cancer Hallmarks tool (Menyhart et al., 2024) and also underwent Gene Ontology Biologic Process (GO-BP) term overrepresentation analysis using the Database for Annotation, Visualization and Integrated Discovery (DAVID) tool (Sherman et al., 2022). A False Discovery Rate (FDR) of <0.05 was considered significant after prior Benjamini-Hochberg adjustment for multiple testing. Details on all database analyses are given in the Supplementary Material.

### 2.2 Cell culture and electroporation

HBL-1, U2932 (both ABC-DLBCL), HT, and WSU-DLCL2 (both GCB-DLBCL) cells were cultivated in RPMI 1640 containing 10% Fetal Calf Serum, 1% Penicillin/Streptomycin, 25 mM HEPES, 1% sodium-Pyruvate, and 0.1% β-Mercaptoethanol at 37°C, 5% CO_2_ in a humid incubator.

For MSI2 knockdown, 1–2*10^6^ cells and 1 nM siPools (unspecific control or MSI2) underwent electroporation using the Neon™ Transfection System (Thermo Fisher Scientific, Waltham, United States). The siPool targeting *MSI2* contained more than 30 different *MSI2*-specific siRNA sequences to reduce off-target effects (Hannus et al., 2014). Electroporation was performed in 3 pulses (10 ms, 1400 V each). Afterwards, cells were transferred into antibiotics-free medium and further cultured.

### 2.3 Flow cytometry

Here, a CyFlow space flow cytometer (Partec, Münster, Germany) was used. CD44 (BD Pharmingen, Franklin Lakes, United States) was measured as described in the manufacturer’s instructions and before (Haiduk et al., 2023). For ALDH, the Aldefluor™ kit (StemCell, Vancouver, Canada) was used per the manufacturer’s manual and as described before (Löblein et al., 2021). Side population was measured with Hoechst 33342 DNA stain (Thermo Fisher Scientific), using an established protocol (Troschel et al., 2021a). If the cell lines showed positivity, further experiments were performed.

Using fluorescence-based cell sorting of the CD44 positive HBL-1 cells, the 10% cells with the highest expression of CD44 were separated from the remainder with a lower CD44 expression. Subsequently, RT-qPCR analyses were performed.

Markers, material and kits are listed in Supplementary Tables S1–S3.

### 2.4 Quantitative real-time PCR (RT-qPCR)

RNA was isolated 48 h after electroporation using the RNeasy®-Kit (Qiagen, Venlo, Netherlands). Reverse transcription was performed using the cDNA Reverse Transcription Kit (Applied Biosystems, Waltham, United States).

RT-qPCR was performed with a Rotor-Gene Q thermocycler (Qiagen), using a TaqMan® Gene Expression Assay (Thermo Fisher Scientific) to evaluate the cycle threshold (ct). The different ct values of mRNA of interest were normalized to 18S rRNA expression. Fold changes were calculated via the 2^−ΔΔct^-method (Löblein et al., 2021). TaqMan probes are listed in Supplementary Table S4.

### 2.5 Western blotting

Protein isolation was performed similar to Greve et al. (2012), and only diverging steps are mentioned. Cells were harvested 72 h after electroporation and around 2–4*10^6^ cells were lysed using RIPA and ultrasound treatment. A Bradford assay was used to measure protein concentrations photometrically. For protein separation 12% Acrylamide Gels or precast gels (BioRad, Hercules, United States) with an acrylamide gradient of 4%–20% were used. For HBL-1 20 µg/lane and for HT 40 µg/lane were applied on the SDS-PAGE. α-Tubulin or β-actin were used as loading control. For antibody data see Supplementary Table S3. Quantitative assessment of protein expression was performed using ImageJ/Fiji.

### 2.6 DNA damage protein activation

Here, a Human DNA Damage Response Phosphorylation Array C1 (RayBiotech Life, Peachtree Corners, United States) was used. 72 h after electroporation, cells were irradiated with 2Gy. Cells were harvested 30 min after irradiation and proteins were isolated. Subsequently, the array was conducted as described by the supplier and analyzed using ImageJ/Fiji.

### 2.7 γH2AX assay

To investigate DNA double-strand breaks after MSI2 knockdown, γH2AX (S139) phosphorylation was measured via flow cytometry after 2Gy irradiation. Staining was performed like before (Greve et al., 2012) with a primary antibody directed at the S139 phosphorylation site and an Alexa Fluor 488 labeled secondary antibody. Cells were harvested after 0 min (without irradiation), and at different timepoints post-irradiation. Again, antibodies are listed in Supplementary Table S3.

### 2.8 Colony formation assay

48 h after electroporation, cells were irradiated with 0, 2, or 4Gy, using a linear accelerator (TrueBeam, Varian, Palo Alto, United States). Afterwards, cells (control vs. MSI2 KD) were transferred into 2 mL of DLBCL-methylcellulose in triplicates for each condition. Cells and methylcellulose were seeded onto cell culture dishes and incubated for 8 (HBL-1) or 6 days (HT) in a humid incubator at standard settings with additional water next to the dishes to inhibit the methylcellulose from drying out. Then, colonies were counted per dish microscopically without staining and fixation of the cells. All dishes of one biological replicate were counted on the same day in order to prevent variability due to ongoing cell growth. Exemplary images of vital colonies in DLBCL-methylcellulose are presented in the Results section. Plating efficacy (PE) and survival fraction (SF) were calculated as follows:

$$\text{PE}=\frac{\text{counted}\;\text{colonies}}{\text{seeded}\;\text{cells}}$$

$$\text{SF}=\frac{\text{PE}\;\left(\text{irradiated}\right)}{\text{PE}\;\left(\text{non}-\text{irradiated}\right)}$$

SFs were compared between control and MSI2 knockdown conditions for radiosensitization analyses.

### 2.9 MTT assay

HBL-1 and HT cells were treated without or with different concentrations of doxorubicin or vincristine directly after electroporation for 96 h. Then MTT (3-(4,5-dimethylthiazol-2-yl)-2,5-diphenyltetrazolium bromide) substrate (Sigma-Aldrich, St Louis, United States) was added for 2 h. The experiment was conducted as described previously (Löblein et al., 2021). The negative control (reagents without cells) was subtracted from the measurements and then each value was normalized to the respective non-chemotherapy treated control. Differences between MSI2 knockdown and control cells were assessed.

### 2.10 Statistics

Statistics were performed using SPSS statistics, Microsoft Excel, R, and GraphPad/Prism 10.4. Experiments were performed at least three times in biological replicates. For statistical analysis, t-tests were performed if not otherwise stated. Values of *p* < 0.05 were considered as statistically significant. For all figures the mean value with each corresponding standard error of the mean (SEM) is displayed.

## 3 Results

### 3.1 Musashi-2 is overexpressed in DLBCL, while Musashi-1 is not relevantly transcribed

**FIGURE 2:**
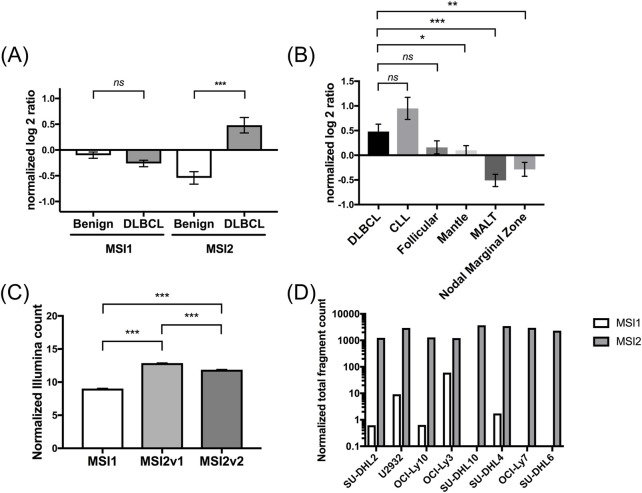
Expression of MSI2 in diffuse large B-cell lymphoma (DLBCL) according to Gene Expression Omnibus (GEO) database analyses. **(A)**: Expression of MSI1 and MSI2 mRNAs compared between non-malignant and DLBCL tissues. MSI1 was non-significantly lower expressed, while MSI2 was significantly overexpressed in DLBCL compared to non-malignant tissues. Comparisons were performed between 22 DLBCL and 13 non-malignant lymphoid tissues from the GSE32018 database. **(B)**: Expression of MSI2 mRNA in different B cell hematological malignancies. DLBCL and CLL showed higher MSI2 mRNA levels compared to other B cell malignancies, including follicular lymphoma, mantle cell lymphoma, Mucosa-associated lymphoid tissue (MALT), and nodal marginal zone lymphoma. Data were again based on secondary analyses from the GSE32018 dataset. **(C)**: Normalized Illumina count of MSI1 and MSI2v1 (MSI2 transcript variant 1) and MSI2v2 (MSI2 transcript variant 2) expression in 1,310 DLBCL samples from the GSE181063 dataset. **(D)**: Expression of MSI1 and MSI2 in 8 established DLBCL cell lines from the GSE50721 dataset. Here, strongly increased normalized counts of MSI2 compared with MSI1 were found. (Significance: *: p ≤ 0.05; **: p ≤ 0.01; ***: p ≤ 0.001. Wilcoxon tests were used to determine statistical significance.)

Considering their close interplay (das Chagas et al., 2020; Troschel et al., 2020; Zhu et al., 2020), we first aimed to establish baseline expression levels of both *MSI1* and *MSI2* in DLBCL. Leveraging the public availability of RNA sequencing data, we performed analyses for *MSI1* and *MSI2* expression levels, finding that compared to benign lymphoid tissue *MSI2* was upregulated in DLBCL tissues while *MSI1* was not (Figure 2A, based on the GSE32018 dataset). Upon further study, we found *MSI2* levels were highest in DLBCL and chronic lymphocytic leukemia (CLL) while expression in other lymphomas was more limited (Figure 2B).

Side-by-side comparisons between *MSI1* and *MSI2* in a set of 1,310 DLBCL patient tissues (GSE181063 dataset) showed that both known *MSI2* transcript variants were strongly overexpressed compared to *MSI1* (*p* < 0.001) while differences between transcript variants were less pronounced (Figure 2C). This finding was confirmed in established DLBCL cell lines (Figure 2D). Some differences were seen regarding expression of *MSI1* and both *MSI2* transcript variants between ABC and GCB DLBCL, but changes in expression levels were marginal (Supplementary Figure S1). Considering these gene expression findings, we decided to focus on MSI2 for further study.

### 3.2 High Musashi-2 levels are associated with reduced OS in DLBCL patients

**FIGURE 3:**
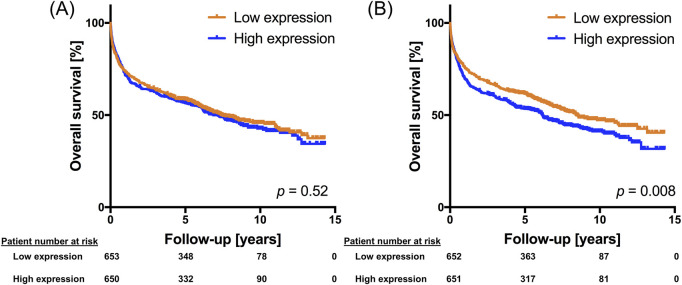
MSI2 expression is associated with overall survival in DLBCL. Kaplan Meier plot using sequencing and patient data from the GSE181063 dataset. MSI2 transcript variant 1 [ILMN_1713088, panel **(A)**] and MSI2 transcript variant 2 [ILMN_1804448, panel **(B)**] expression was dichotomized at the median expression into high-expressing and low-expressing DLBCL tissues. A log rank test was performed to determine statistical significance.

Given conflicting data on the prognostic relevance of *MSI2* in solid tumor entities (Jiang et al., 2022; Li et al., 2022) we next investigated the interplay between *MSI2* expression and OS (Figures 3A,B). OS was significantly longer in patients with below-median *MSI2* transcript variant 2 levels compared to above-median expression in the GSE181063 dataset (Figure 3B, *p* = 0.008), while no differences were seen for transcript variant 1 (Figure 3A, *p* = 0.52). No difference in *MSI2* expression was seen between males and females (*p* = 0.89) and we also did not observe any association between *MSI2* expression and age (*p* = 0.46). Conversely, patients treated in curative intent tended to have lower *MSI2* expression than those treated in non-curative intent (*p* = 0.025, Supplementary Table S5).

We next performed multivariable Cox regression analyses in a total of four large DLBCL gene expression datasets. Besides *MSI2* expression, we included established risk factors IPI score and DLBCL subtype in the models, if available in the datasets. Three of four datasets confirmed a significant negative association between *MSI2* expression and OS (Table 1).

**TABLE 1:** Multivariable models for overall survival (OS) containing the most common Musashi-2 transcript variant 1 or transcript variant 2 reads, or, if no difference between transcript variants was made during sequencing, the most common overall Musashi-2 read in four publicly available DLBCL datasets. Additionally, subtype and IPI score were included. The number of patients included in the multivariable model relative to the number of all patients in the dataset (= inclusion rate) is also given for each dataset. Exclusions were solely due to unavailability of data.

	GSE181063				GSE31312		GSE10846		GSE87371	
Transcript for MSI2	ILMN_1713088 (MSI2 transcript variant 1)		ILMN_1804448 (MSI2 transcript variant 2)		225240_s_at		225240_s_at		225240_s_at	
Number of patients/inclusion rate	1303/1311 (99.4%)		1303/1311 (99.4%)		424/498 (85.1%)		313/420 (74.5%)		221/223 (99.1%)	
	HR	*p*	HR	*p*	HR	p	HR	*p*	HR	*p*
MSI2	1.04 (0.94–1.14)	0.46	1.10 (1.03–1.18)	**0.008**	6.90 (1.57–30.22)	**0.010**	1.02 (0.83–1.26)	0.82	1.54 (1.05–2.22)	**0.024**
Subtype										
ABC	Ref 1.00		Ref 1.00		Ref 1.00		Ref 1.00		Ref 1.00	
GCB	0.54 (0.45–0.65)	**<0.001**	0.45 (0.45–0.65)	**<0.001**	0.64 (0.45–0.89)	**0.009**	0.42 (0.28–0.63)	**<0.001**	0.48 (0.24–0.92)	**0.02**
Unknown/ Unclassified/ Otherwise classified	0.71 (0.59–0.85)	**<0.001**	0.70 (0.58–0.84)	**<0.001**	1.03 (0.60–1.76)	0.92	0.53 (0.32–0.89)	**0.017**	0.58 (0.27–1.25)	0.17
IPI score	limited availability (<70%)^a^				1.74 (1.53–1.99)	**<0.001**	1.68 (1.44–1.95)	**<0.001**	2.65 (1.87–3.75)	**<0.001**

^a^ The IPI score was unavailable in more than 30% of patients in the GSE181063 dataset and disproportionately in those with a very limited survival (p < 0.001 in log rank testing) and increased MSI2 transcript variant 2 expression (p = 0.01 in Mann Whitney U testing), potentially introducing bias for modeling, precluding its use in a multivariable model.

Bold values indicate the significant p-values.

### 3.3 Musashi-2 is co-expressed with markers of DNA repair and proliferation

We next aimed to understand the molecular profile associated with *MSI2* expression in DLBCL. A query of the UALCAN tool for genes co-expressed with *MSI2* within the TCGA-DLBC dataset returned a list of 7,148 genes (Supplementary Table S6).

**FIGURE 4:**
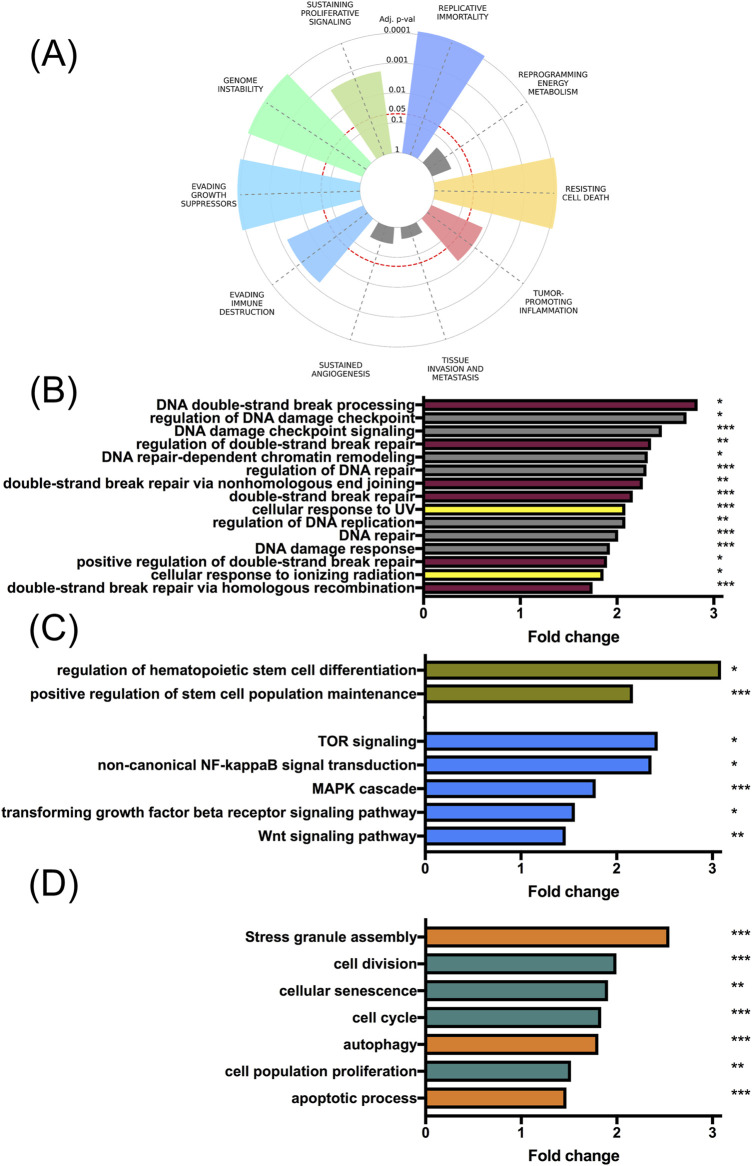
MSI2 is co-expressed with multiple cancer hallmarks. Using the “The University of ALabama at Birmingham CANcer data analysis Portal” (UALCAN), genes co-expressed with MSI2 within the “Lymphoid Neoplasm Diffuse Large B-cell Lymphoma” from “The Cancer Genome Atlas” (TCGA-DLBC) were queried. **(A)**: Multiple cancer hallmarks are overexpressed among genes co-expressed with MSI2, including genome instability. **(B)**: GO term analyses indicated a substantial number of DNA repair-related gene ontology (GO) terms overrepresented among genes co-expressed with MSI2 in DLBCL. DNA double-strand break-related GO terms (dark red), and radiation-associated terms (yellow) are highlighted. **(C)**: GO term analyses similarly indicated the overrepresentation of pathways associated with stemness. Stemness GO terms (green) and stemness-associated signaling pathways (blue) are highlighted. **(D)**: Pathways associated with proliferation and apoptosis regulation were similarly co-expressed. Proliferation- and cell cycle-associated GO terms (green) and apoptosis-, stress- and autophagy-related GO terms (orange) are highlighted. Significance of GO term overrepresentation was determined using the Benjamini Hochberg procedure.

We first submitted this list to an enrichment tool for cancer hallmarks (Menyhart et al., 2024). We found multiple hallmarks overrepresented among our gene list including genome instability [odds ratio (OR) 3.72], replicative immortality (OR 3.22), and resisting cell death (OR 1.92, Figure 4A, full data in Supplementary Table S7). In GO-BP term analysis using the DAVID tool, numerous DNA repair-associated terms were overrepresented among MSI2 co-expressed genes (Figure 4B, DNA double-strand break-associated GO terms in dark red, and radiation-associated GO terms in yellow). This included important radiotherapy-related signaling (e.g., “DNA damage response,” “regulation of double-strand break repair,” and “cellular response to ionizing radiation”). Additionally, stemness-related (Figure 4C, stemness GO terms in green and stemness-associated pathway GO terms in blue) and proliferation- and apoptosis-associated signaling (Figure 4D, proliferation and cell cycle-related GO terms in green, and apoptosis-, stress-, and autophagy-linked GO terms in orange) was also co-expressed. For all co-expressed terms see Supplementary Table S8.

As these data suggested an association between *MSI2* expression and therapy-relevant signaling, most prominently DNA maintenance, we decided to target MSI2 in DLBCL to assess subsequent changes in treatment response. We chose HBL-1, an ABC-DLBCL cell line, and HT, a GBC-DLBCL cell line, as models. Electroporation-mediated siRNA-based knockdown resulted in satisfactory knockdown success in both cell cultures (*p* < 0.01, Supplementary Figure S2).

### 3.4 Musashi-2 knockdown attenuates stemness- and DNA repair-related signaling

**FIGURE 5:**
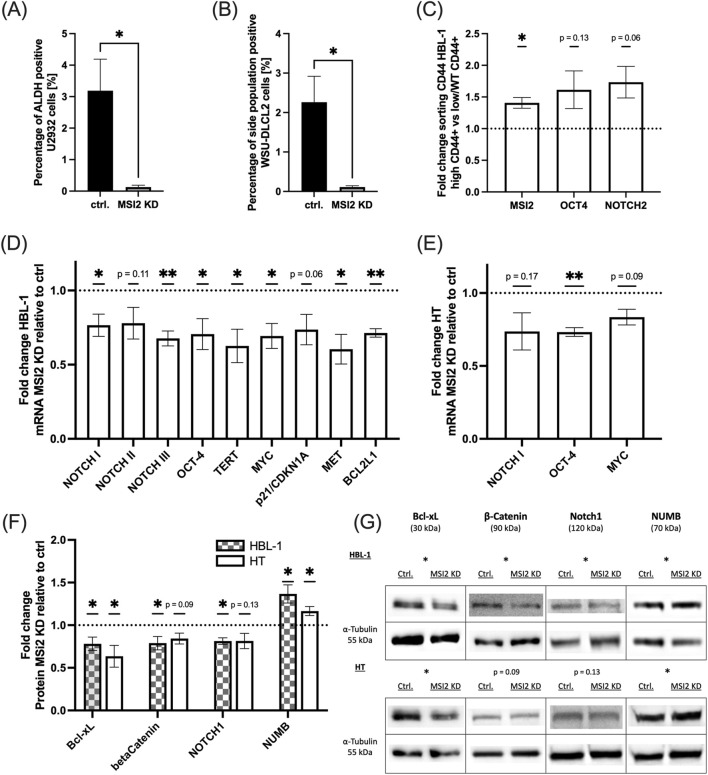
MSI2 knockdown attenuates stemness-related signaling. **(A)**: ALDH positive U2932 DLBCL cells as measured via flow cytometry with and without MSI2 knockdown. **(B)**: Side population WSU-DLCL2 cells as measured via flow cytometry with and without MSI2 knockdown. **(C)**: mRNA expression of MSI2, Oct4, and NOTCH2 in wildtype HBL-1 cells enriched for CD44 via fluorescence-based cell sorting. **(D,E)**: Changes in mRNA levels of stemness markers after MSI2 knockdown as measured via qPCR in HBL-1 **(D)** and HT **(E)** cells. **(F,G)**: Corresponding western blot analyses with representative blots in **(G)**. (Significance: *: p ≤ 0.05; **: p ≤ 0.01; ***: p ≤ 0.001; All experiments were performed in at least three independent repetitions. T tests were used to determine statistical significance.)

We first assessed stemness-associated markers flow cytometrically. As expression varied substantially in DLBCL cells, we tested two additional cell lines (U2932, WSU-DLCL2) for ALDH, side population, and CD44. Of all analyzed cell lines, only U2932 was positive for ALDH, only WSU-DLCL2 showed a relevant proportion of side population cells, and only HBL-1 showed positivity for CD44 (representative images of flow cytometric histograms showing negativity or positivity in Supplementary Figures S3, S4). MSI2 knockdown reduced ALDH positive cell fractions in U2932 to less than a tenth of their previous levels (*p* = 0.04, Figure 5A), with similar results regarding side population cells in WSU-DLBCL2 (*p* = 0.02, Figure 5B). CD44 levels were only slightly reduced in HBL-1 (*p* = 0.06, Supplementary Figure S5). Conversely, in untreated, sorted CD44-enriched HBL-1 cells, *MSI2* mRNA levels were roughly 50% increased (*p* = 0.02), as were stemness markers *Oct4* (*p* = 0.13) and *NOTCH-2* (*p* = 0.06, Figure 5C).

We next performed qPCR analyses for *NUMB/NOTCH* signaling elements as well as *Oct4*, *TERT* and other stemness-associated genes in HBL-1 cells and HT, finding a consistent downregulation of signaling after MSI2 knockdown (Figures 5D,E). Confirmatory western blot analyses of NUMB, a NOTCH pathway repressor, as well as stemness markers NOTCH1, beta catenin, and Bcl-xL in both HBL-1 and HT cells demonstrated a loss of stemness-related signaling (Figure 5F, representative blots in Figure 5G, exemplary biological replicates in Supplementary Figure S6).

**FIGURE 6:**
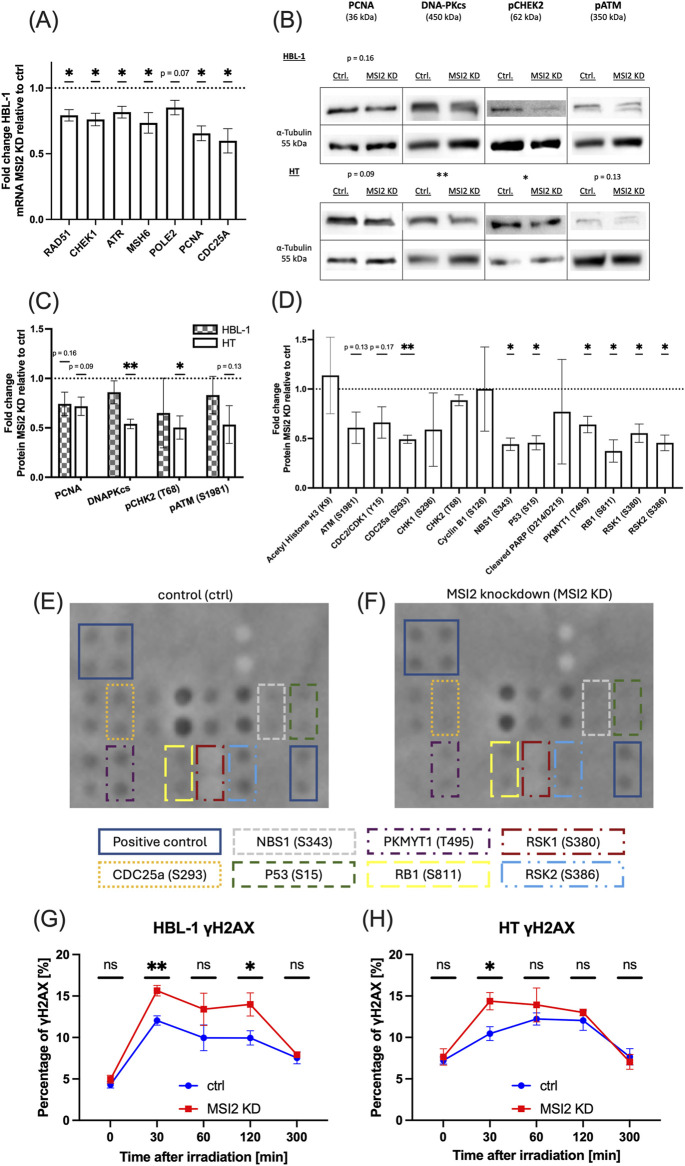
Radiation-induced DNA damage response and DNA double-strand breaks. **(A)**: qPCR analyses for DNA repair-related genes in MSI2 knockdown HBL-1 cells compared to wildtype controls. **(B,C)**: Western blot studies of DNA repair-associated proteins in MSI2 knockdown HBL-1 and HT cells compared to wildtype controls. Representative blots are shown in **(B)**, while statistical analysis of all performed blots are shown in **(C)**. **(D)**: DNA damage protein phosphorylation 30 min after 2Gy irradiation in MSI2 knockdown HT DLBCL cells compared to similarly irradiated wildtype cells analyzed in biological replicates. **(E,F)**: Representative image of phosphorylated proteins in wildtype condition **(E)** and after MSI2 knockdown **(F)**, 30 min after irradiation with 2 Gy. Significantly altered proteins and positive controls are marked (see remaining biological replicates in Supplementary Figure S7). **(G,H)**: Double-strand breaks as measured via γH2AX in HBL-1 **(G)** and HT **(H)** DLBCL cells either after MSI2 knockdown (red) or in wildtype conditions (blue). γH2AX was measured flow cytometrically before (t = 0) and at 30, 60, 120, and 300 min after irradiation with 2 Gy. (Significance: *: p ≤ 0.05; **: p ≤ 0.01; ***: p ≤ 0.001; All experiments were performed in at least three independent repetitions. T tests were used to determine statistical significance.).

We additionally found numerous DNA repair-related genes downregulated in qPCR analyses in HBL-1 cells (Figure 6A). After confirming moderate downregulation of four pivotal DNA repair-associated proteins, namely PCNA, DNA-PKcs, and phosphorylated versions of CHEK2 (pCHEK2) and ATM (pATM) in western blot analyses for both cell cultures (Figure 6C, representative blots in Figure 6B, exemplary biological replicates in Supplementary Figure S6), we performed a DNA Damage Response Phosphorylation Array in HT cells after inducing DNA damage with 2Gy irradiation in biological replicates. We found DNA damage repair activation to be severely repressed in MSI2 knockdown cells (Figure 6D, representative images in Figures 6E,F, additional images of biological replicates in Supplementary Figure S7). We then hypothesized that repressed DNA repair capabilities might increase DNA instability, especially after DNA-targeted treatment. Consequently, DNA double-strand break levels, as measured flow cytometrically via positivity for phosphorylated H2AX, were increased after a radiation dose of 2Gy in both cell cultures. Notably, no differences were seen before genotoxic treatment (at t = 0, Figures 6G,H).

### 3.5 MSI2 knockdown sensitizes DLBCL cells to radiotherapy and DNA-targeted chemotherapy

**FIGURE 7:**
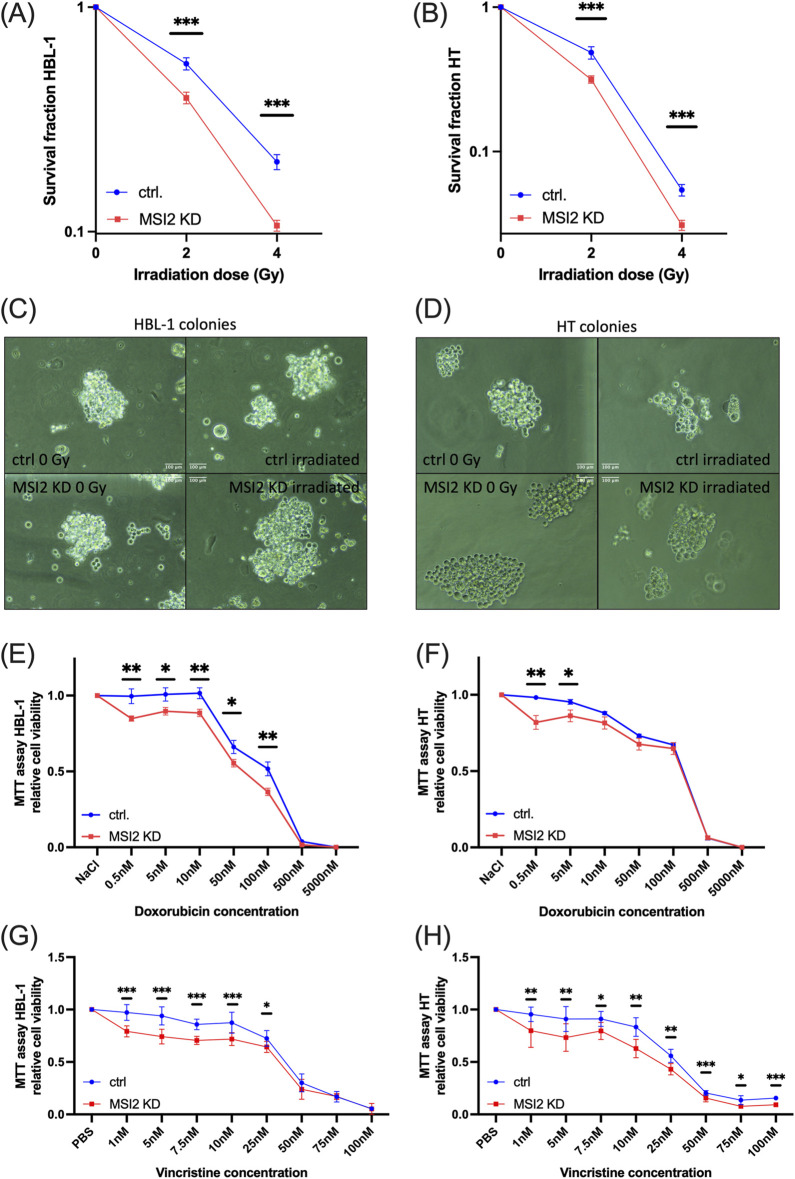
MSI2 knockdown sensitizes DLBCL cells to irradiation, doxorubicin, and vincristine chemotherapy. **(A,B)**: Colony formation assay in HBL-1 **(A)** and HT **(B)** DLBCL cells after MSI2 knockdown (red) or in wildtype condition (blue). Cells were treated with 2 or 4 Gy irradiation doses. **(C,D)**: Representative colonies of HBL-1 **(C)** and HT **(D)** cells with the different conditions. Scale bar 100 µm length. **(E,F)**: Cell viability assay in HBL-1 **(E)** and HT **(F)** DLBCL cells after MSI2 knockdown (red) or in wildtype condition (blue). Cells were treated with different concentrations of doxorubicin. **(G,H)**: Cell viability assay in HBL-1 **(G)** and HT **(H)** DLBCL cells after MSI2 knockdown (red) or in wildtype condition (blue). Cells were treated with different concentrations of vincristine. (Significance: *: p ≤ 0.05; **: p ≤ 0.01; ***: p ≤ 0.001; All experiments were performed in at least three independent repetitions. T tests were used to determine statistical significance.)

After demonstrating increased radiation-induced genotoxicity after MSI2 knockdown, we hypothesized that cell survival would be adversely affected by combined treatment. We found a loss in clonogenic ability after MSI2 knockdown and irradiation doses of 2 and 4Gy, suggesting that MSI2 knockdown sensitizes DLBCL cells to irradiation (Figures 7A,B). At 4Gy, clonogenic ability was reduced by nearly 50% in MSI2 knockdown cells compared to similarly irradiated controls in both cell cultures. Colonies showed subtle changes in cell morphology after MSI2 knockdown and irradiation including more singularized cells and moderate cellular swelling, making MSI2 knockdown cell colonies appear visually larger despite being comprised of fewer cells (Magnification ×200, Figures 7C,D).

We finally subjected MSI2 knockdown cells to DNA-intercalating doxorubicin, a standard part of first-line R-CHOP therapy. While effects were moderate, we also found a therapy-sensitizing response (Figures 7E,F). Additionally, cells were treated with varying concentrations of vincristine, a chemotherapeutic agent also included in the R-CHOP regimen. Chemosensitizing effects were observed, with a more pronounced response in HT cells (Figures 7G,H). Notably, MSI2 knockdown cells exhibited most markedly reduced viability at lower concentrations of both chemotherapeutic agents.

## 4 Discussion

In our study we found that (1) MSI2 is overexpressed and prognostically significant in DLBCL, (2) MSI2 expression interacts with multiple signaling pathways, including stemness and DNA damage repair in DLBCL, and (3) MSI2 knockdown sensitizes DLBCL to chemotherapy and irradiation.

### 4.1 MSI2 is overexpressed and prognostically significant in DLBCL

Our database analyses demonstrate that *MSI2* is overexpressed in DLBCL while *MSI1* is not relevantly expressed, similar to findings in AML (Kharas et al., 2010). Among different lymphoid neoplasms, CLL and DLBCL showed the most prominent upregulation of *MSI2*. Accordingly, MSI2 has been the focus of a comprehensive investigation in CLL demonstrating its involvement in tumorigenicity and its association with reduced OS (Palacios et al., 2021). Intriguingly, despite more modest expression in our analyses, MSI2 has also been characterized as a pivotal tumorigenic marker and a marker of reduced OS in mantle cell lymphoma (Sureda-Gómez et al., 2023). The finding of MSI2 overexpression is significant as it suggests that MSI2 targeting may disproportionately affect MSI2 high-expressing tumor cells, as has similarly been proposed–and validated–for triple-negative breast cancer (Brücksken et al., 2025).

Our study establishes that increased *MSI2* expression is associated with reduced OS in DLBCL. Our univariable analyses only identified the less-common transcript variant 2, one of two splicing isoforms, as a prognostic factor in a large DLBCL cohort. At this time, very limited data is available on different MSI2 transcript variants, though a study in triple-negative breast cancer suggests possible isoform-specific roles (Li et al., 2020). However, our multivariable analyses in additional datasets that did not allow for discrimination between transcript variants convincingly demonstrated that overall *MSI2* expression was prognostically relevant independent of IPI score and DLBCL subtype. Importantly, *MSI2* expression was not associated with patient age or gender, indicating that its expression is likely a tumor-specific rather than a patient-specific characteristic.

In accordance with our results, limited data from other hematologic malignancies also identified high MSI2 expression as a negative prognostic marker in acute myeloid leukemia (Byers et al., 2011), acute lymphoblastic leukemia (Mu et al., 2013), chronic lymphocytic leukemia (Palacios et al., 2021), and aggressive mantle cell lymphoma (Sureda-Gómez et al., 2023). MSI2 has also been found to be overexpressed and a marker of negative prognosis in many solid malignancies (Jiang et al., 2022). Notable exceptions include clear cell renal cell carcinoma (Li et al., 2022) and triple-negative breast cancer, where conflicting data exist (Li et al., 2020; Troschel et al., 2020; Haiduk et al., 2023; Sicking et al., 2023).

### 4.2 MSI2 interacts with multiple signaling pathways, including stemness and DNA damage repair in DLBCL

Response to first-line immunochemotherapy is the key determinant of outcome in DLBCL (Coiffier and Sarkozy, 2016). Hence, after finding *MSI2* levels to be prognostically relevant in DLBCL, we hypothesized their involvement in therapy resistance.

Analyses of genes co-expressed with *MSI2* further supported this assumption, suggesting involvement in multiple cancer hallmarks, mainly DNA maintenance and cell death resistance. In GO-BP term analyses, we found two prominent therapy resistance-associated signals:

#### 4.2.1 Stemness-related signaling

First established as a stem cell marker (Sugiyama-Nakagiri et al., 2006), MSI2 has long been implicated in stemness: MSI2 may increase NOTCH signaling by directly binding and downregulating the NOTCH repressor NUMB (Nishimoto and Okano, 2010). In different cell types, MSI2 has also been connected to wnt, MYC (Kharas et al., 2010), TGF beta (Kudinov et al., 2016), and NF-κB signaling (Fujiwara et al., 2016), all of which have been associated with stemness. Some of these pathways were co-expressed with *MSI2* in our gene expression analyses, possibly suggesting similar relationships for DLBCL. Our *in vitro* knockdown analyses confirmed that loss of MSI2 abrogates stemness. Intriguingly, markers lost after MSI2 knockdown including ALDH (Song et al., 2014), side population (Chen et al., 2020), and NOTCH signaling (Hartert et al., 2021) have each been implicated in DLBCL therapy resistance, supporting further assessment of treatment response.

#### 4.2.2 DNA repair-related signaling

DNA repair-related signaling was strongly co-expressed with *MSI2*. Accordingly, DNA repair was downregulated after MSI2 knockdown. Even though several effects in western blotting and DNA damage phosphorylation array analysis were moderate, the reduction in most DNA repair associated pathways emphasizes the influence of MSI2 on DNA repair-related signaling. This is in line with findings in lung cancer implicating MSI2 in DNA damage repair (Qu et al., 2024; Barber et al., 2025) and in triple-negative breast cancer regarding double knockdown of MSI1 and MSI2 (Troschel et al., 2020). Recent unpublished work (Bychkov et al., 2024) suggests that MSI2 may directly bind the ATM mRNA which may explain the observed reduction in pATM levels following MSI2 knockdown in our study, along with the associated pathway effects. Beyond ATM, the interaction between MSI proteins and DNA-PKcs—a critical protein in the repair of double-strand breaks—has also been well-documented (de Araujo et al., 2016; Troschel et al., 2020; Falke et al., 2022). Besides pATM and DNA-PKcs, we also found downstream signaling pathways of RAD51, CHK1, and phosphorylated CHK2, as well as several other DNA damage response proteins to be affected, indicating effects of MSI2 KD on multiple levels of DNA damage response-related signaling pathways. We consequently found increased radiotherapy-induced DNA double-strand breaks after MSI2 knockdown, especially in shorter time periods after irradiation treatment. Intriguingly, we found substantial effects despite only limited MSI2 knockdown efficiency, suggesting the potential for improved effects in more complete knockout approaches. Importantly, however, the knockdown efficiency we found is not substantially worse compared to a MSI2 knockdown study in CLL (Palacios et al., 2021).

Overall, our analyses indicated the potential of combining DNA-targeted therapy with MSI2 knockdown.

### 4.3 MSI2 knockdown sensitizes DLBCL to chemotherapy and irradiation

We tested three DNA-targeted therapies in combination with MSI2 knockdown:

Knockdown of MSI2 led to a modest chemosensitization to doxorubicin, a DNA-intercalating drug, in the HBL-1 cell line, as well as at low doses in the HT cell line. Supporting this, chemosensitization of hematopoietic malignancies after MSI2 silencing was also observed in acute myeloid leukemia regarding daunorubicin (Han et al., 2015), and mantle cell lymphoma regarding doxorubicin (Sureda-Gómez et al., 2023). Doxorubicin was tested because it is part of current first-line DLBCL therapy, R-CHOP (Sehn and Salles, 2021), and doxorubicin-resistant DLBCL cells were previously shown to overexpress stemness properties (Ryu et al., 2017).

Effects may have either been conferred via reduced DNA damage repair which is crucial for efficacy of doxorubicin (Pfitzer et al., 2019). Alternatively, Bcl-xl, a stemness marker lost after MSI2 knockdown has been associated with doxorubicin resistance in breast cancer (Fiebig et al., 2006). Notably, direct Bcl-xl inhibition has been proposed for DLBCL treatment (Klanova et al., 2016).

In addition to doxorubicin, we also tested vincristine, another important component of the R-CHOP regimen. While vincristine primarily targets microtubule assembly, it has also been implicated in DNA damage induction and impaired DNA synthesis (Tsukamoto and Kojo, 1989; Zhang and Sun, 1992). MSI2 knockdown meaningfully sensitized DLBCL cells to vincristine treatment. This increased chemosensitivity may be attributed to reduced levels of DNA damage response proteins following MSI2 knockdown, which could increase vincristine efficacy. Previous studies suggest that high DNA repair capacity may attenuate vinca alkaloid effectiveness (Ehrhardt et al., 2013), and that vincristine disrupts intracellular trafficking of DNA repair proteins (Poruchynsky et al., 2015), supporting a synergistic effect between MSI2 knockdown and vincristine treatment.

A recent study in DLBCL also implicated MSI2 in the resistance to PRMT5 inhibitors, suggesting that Musashi may also target other substance groups (Erazo et al., 2022).

Intriguingly, the strongest sensitizing effects were observed when combining MSI2 targeting with ionizing radiation, a mainstay of DLBCL treatment (Oertel et al., 2023). While previous studies have demonstrated that MSI1 knockdown (de Araujo et al., 2016; Lin et al., 2018; Troschel et al., 2021b; Falke et al., 2022) or dual MSI1/MSI2 targeting (Troschel et al., 2020; Löblein et al., 2021) sensitize solid malignancies to radiotherapy, data on the radiation relevance of MSI2 remain rare (Qu et al., 2024) and neither approach has previously been tested in hematologic malignancies. Our study suggests that MSI2 knockdown compromises DNA repair, increasing DNA double-strand breaks and reducing cell survival. Our γH2AX data showed a clear increase in DNA double-strand breaks immediately after irradiation in MSI2 knockdown compared to control cells while no differences were observed in unirradiated cells or at later timepoints after irradiation. We interpret these findings to suggest that MSI2 depletion alone may compromise DNA repair capacity, although not to a degree sufficient to alter baseline levels of DNA damage. In contrast, under conditions of genotoxic stress, such as ionizing radiation, the deficiency becomes functionally significant, leading to phenotypic differences. The absence of differences in H2AX phosphorylation at later time points post-irradiation may not necessarily indicate successful DNA repair in MSI2 knockdown cells but could instead reflect early cell death or depletion of DNA repair capacity in surviving cells. Importantly, the H2AX assays does not measure DNA double-strand breaks directly but the H2AX phosphorylation of these lesions, an ATM-dependent (Burma et al., 2001) process. However, we have shown that pATM is downregulated in MSI2 KD cells, suggesting this method may actually underestimate the level of DNA double-strand breaks in MSI2 KD cells. Taken together with our observation that cell survival is significantly reduced in irradiated MSI2 KD cells, these findings support the conclusion that the observed decrease in H2AX phosphorylation over time is not indicative of delayed but successful DNA repair, but rather a consequence of cell death due to inadequate repair mechanisms or exhaustion of ATM-dependent H2AX phosphorylation processes.

MSI2 inhibitors, which have previously been tested *in vitro* in hematologic malignancies (Minuesa et al., 2019; Sureda-Gómez et al., 2023), should therefore be assessed in combination with radiation treatment. Although *MSI2* overexpression in DLBCL indicates that MSI2 inhibitors may preferentially target tumor cells, future *in vivo* studies are essential to evaluate potential effects on normal tissues. Elucidating radiation resistance mechanisms is clinically relevant as radiotherapy is increasingly used for relapsed or refractory DLBCL (Ng et al., 2018), where radioresistance is presumed higher (Aref et al., 1999). Hence, targeting the DNA damage response in lymphoma to increase efficacy of genotoxic therapy is receiving increasing attention (Carrassa et al., 2020; Mansoor et al., 2024). These findings of our study are well in line with data from other recently published studies, which also show a clear association between the Musashi proteins and the modulation of the DNA damage response in lung cancer and triple-negative breast cancer (Qu et al., 2024; Barber et al., 2025; Brücksken et al., 2025).

Overall, our findings suggest that MSI2, a negative prognostic marker in DLBCL, modulates genome stability via DNA repair activation. The combination of MSI2 knockdown and DNA-directed therapy such as radiotherapy increases DLBCL treatment efficacy *in vitro*, uncovering a mechanism to potentially mitigate therapy resistance.

## Data Availability

The raw data supporting the conclusions of this article will be made available by the authors, without undue reservation.

## Abbreviations

DLBCL, Diffuse large B-cell lymphoma; MSI, Musashi RNA binding protein, either 1 or 2; GCB, Germinal center B-cell type, subtype of diffuse large B-cell lymphoma; ABC, Activated B-cell type, subtype of diffuse large B-cell lymphoma; R-CHOP, Immunochemotherapy consisting of Rituximab, cyclophosphamide, doxorubicin, vincristine, prednisolone; CSCs, Cancer stem cells; UTR, Untranslated regions of mRNA; OS, Overall survival; IPI, International Prognostic Index; HR, Hazard ratio; CI, Confidence interval; TCGA, The Cancer Genome Atlas; TCGA-DLBC, The Cancer Genome Atlas database for Lymphoid Neoplasm Diffuse Large B-cell Lymphoma; UALCAN, The University of ALabama at Birmingham CANcer data analysis portal; GO, Gene Ontology; GO-BP, Gene Ontology Biologic Process; FDR, False Discovery Rate; siRNA, small interfering RNA; MTT, 3-(4,5-dimethylthiazol-2-yl)-2,5-diphenyltetrazolium bromide, reagent for cell viability assay; SEM, Standard Error of the Mean; OR, Odds ratio; AML, Acute myeloid leukemia; CLL, Chronic lymphocytic leukemia.
