# Supplementary material for: Prognostic marker Musashi-2 modulates DNA damage response and radioresistance in diffuse large B-cell lymphoma
Source: Front Cell Dev Biol. 2025 Aug 6;13:1575483. doi: 10.3389/fcell.2025.1575483 (PMC12364875; doi:10.3389/fcell.2025.1575483)
Supplement: Supplementary file 1 [file DataSheet3.pdf]

Supplementary Figure 1: MSI1, MSI2 transcript variant 1 (MSI2v1) and MSI2 transcript variant 2 (MSI2v2) expression in DLBCL patients, stratified by subgroup.

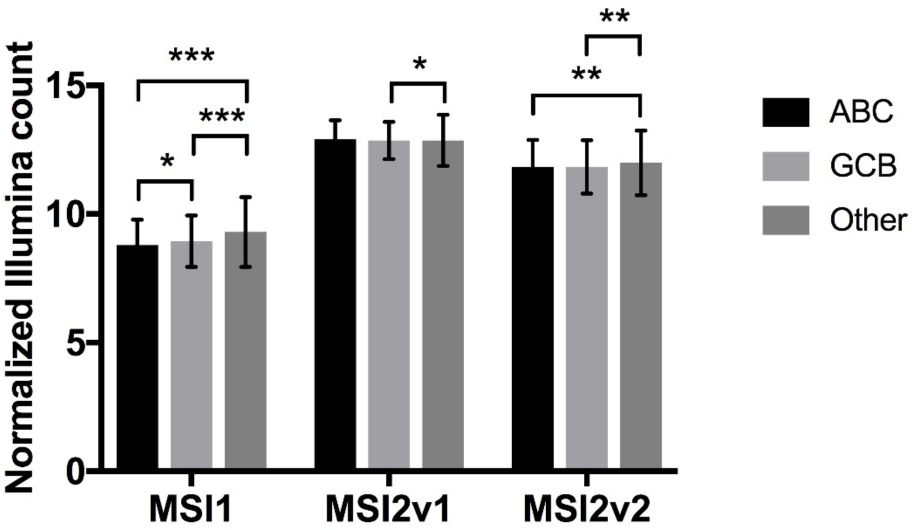

Supplementary Figure 1: MSI1, MSI2 transcript variant 1 (MSI2v1) and MSI2 transcript variant 2 (MSI2v2) expression in DLBCL patients, stratified by subgroup. DLBCL patients were divided into activated B-cell (ABC, n = 345), germinal center B-cell (GCB, n = 517), and a third category (n = 448) comprising all other patients (unclassified or other subgroups). (Significance: *: $p \leq 0.05$; **: $p \leq 0.01$; ***: $p \leq 0.001$)

Supplementary Figure 2: MSI2 knockdown verification

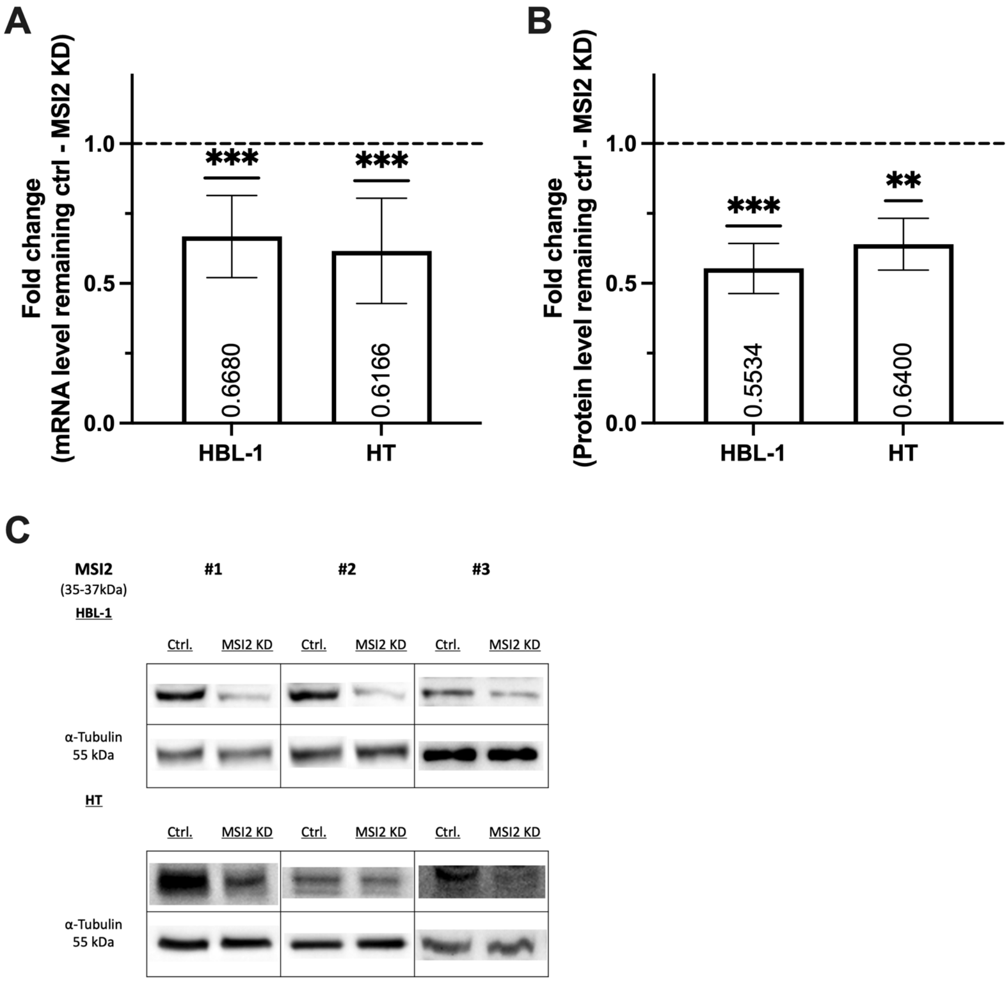

Supplementary Figure 2: MSI2 knockdown verification. (A) & (B): MSI2 levels in HBL-1 and HT DLBCL cells after electroporation-based knockdown using siPool sequences. qPCR analyses (A) and Western Blot (B) were performed. **(C)**: Western Blot data. (Significance: *: $p \leq 0.05$; **: $p \leq 0.01$; ***: $p \leq 0.001$; All experiments were performed in at least three independent repetitions)

Supplementary Figure 3: CD44 stemness-related marker positivity

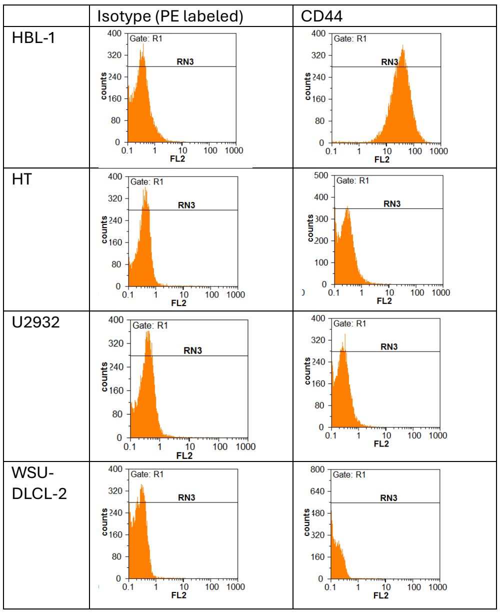

Supplementary Figure 3: CD44 stemness-related marker positivity. Depicted are flow cytometric measurements of stemness related cellular surface marker CD44. If a cell line showed positivity, analysis of the marker was performed after MSI2 KD. Here, only HBL-1 showed a relevant positivity for stemness marker CD44 and was therefore used for analyses.

Supplementary Figure 4: Flow cytometry analysis for side population and ALDH positivity.

	Side population – Verapamil (suppressed)	Side Population (non-suppressed)	ALDH – DEAB (inhibitor)	ALDH stain
HBL-1	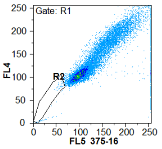 R2 = 0.31%	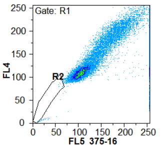 R2 = 0.10%	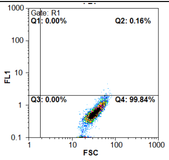	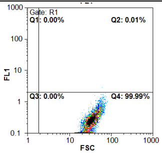
HT	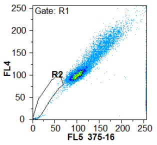 R2 = 0.39%	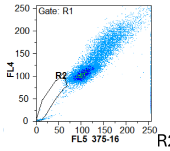 R2 = 0,50%	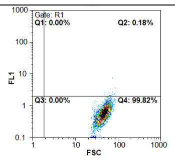	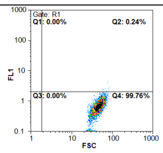
U2932	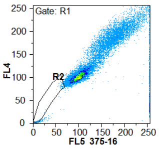 R2 = 0.87%	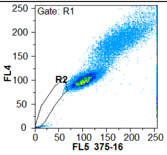 R2 = 0.67%	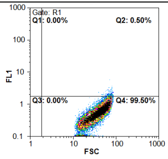	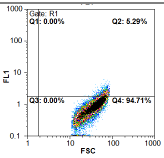
WSU-DLCL-2	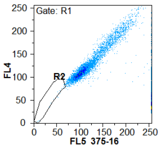 R2 = 0.20%	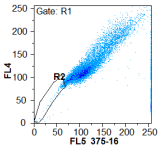 R2 = 1.52%	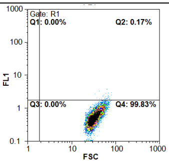	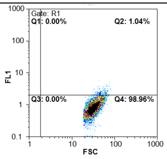

Supplementary Figure 4: Flow cytometry analysis for side population and ALDH positivity. All cell lines were tested for their positivity regarding side population and ALDH. Here, U2932 wildtype cells showed to be relevantly positive for ALDH at a percentage of 5%. WSU-DLCL-2 cell line was relevantly positive for side population, while the observed slight difference in ALDH positivity compared to the ALDH-repressed (DEAB) cells was not deemed relevant. Thus, experiments were performed with U2932 cells regarding ALDH positivity and with WSU-DLCL-2 cells regarding side population positivity.

Supplementary Figure 5: CD44 levels in HBL-1 cells after MSI2 knockdown

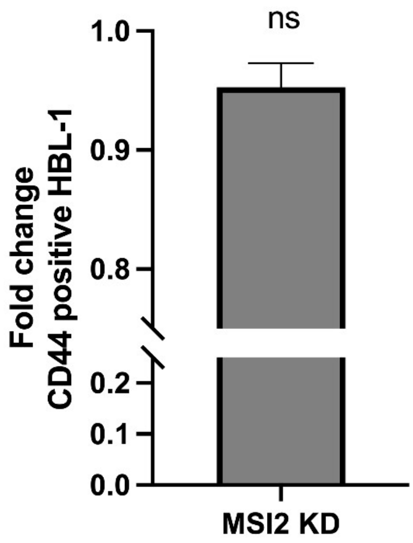

Supplementary Figure 5: CD44 levels in HBL-1 cells after MSI2 knockdown. Data from Msi2 siRNA treated cells are presented relative to control siRNA treated cells.

Supplementary Figure 6: Exemplary biological replicates of western blotting experiments

HBL-1	Biological Replicate 1<br>ctrl: left<br>MSI2 KD: right	Biological Replicate 2<br>ctrl: left<br>MSI2 KD: right	Biological Replicate 3<br>ctrl: left<br>MSI2 KD: right
Bcl-xL<br>(30 kDa)<br>α-Tubulin<br>(55 kDa)	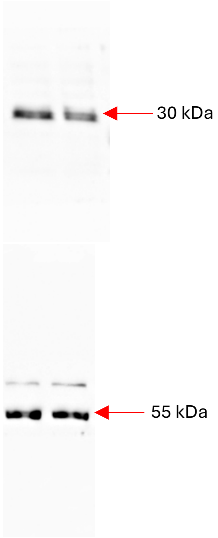	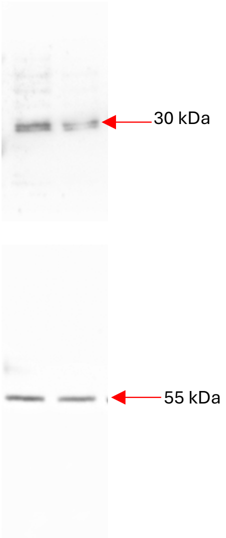	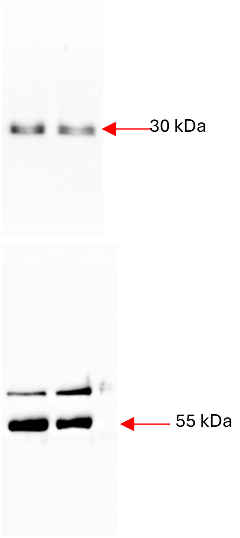
β-Catenin<br>(90 kDa)<br>α-Tubulin<br>(55 kDa)	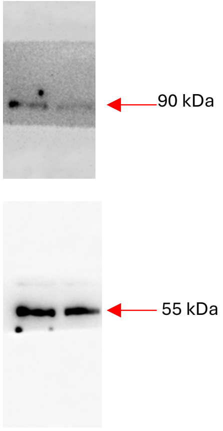	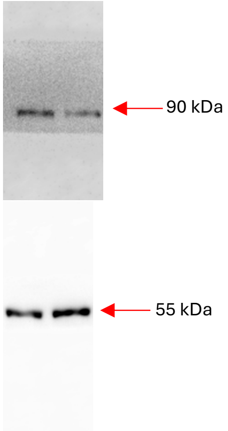	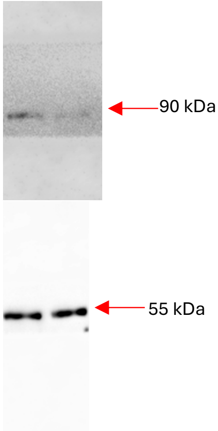
NOTCH-1<br>(120 kDa)<br>α-Tubulin<br>(55 kDa)	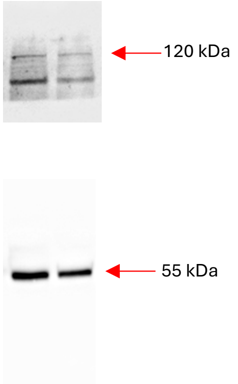	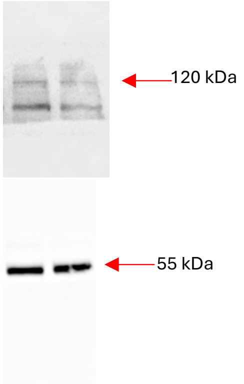	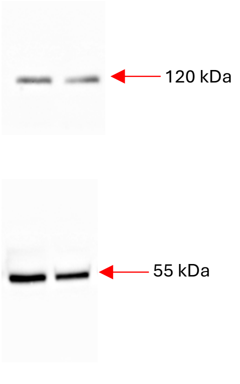

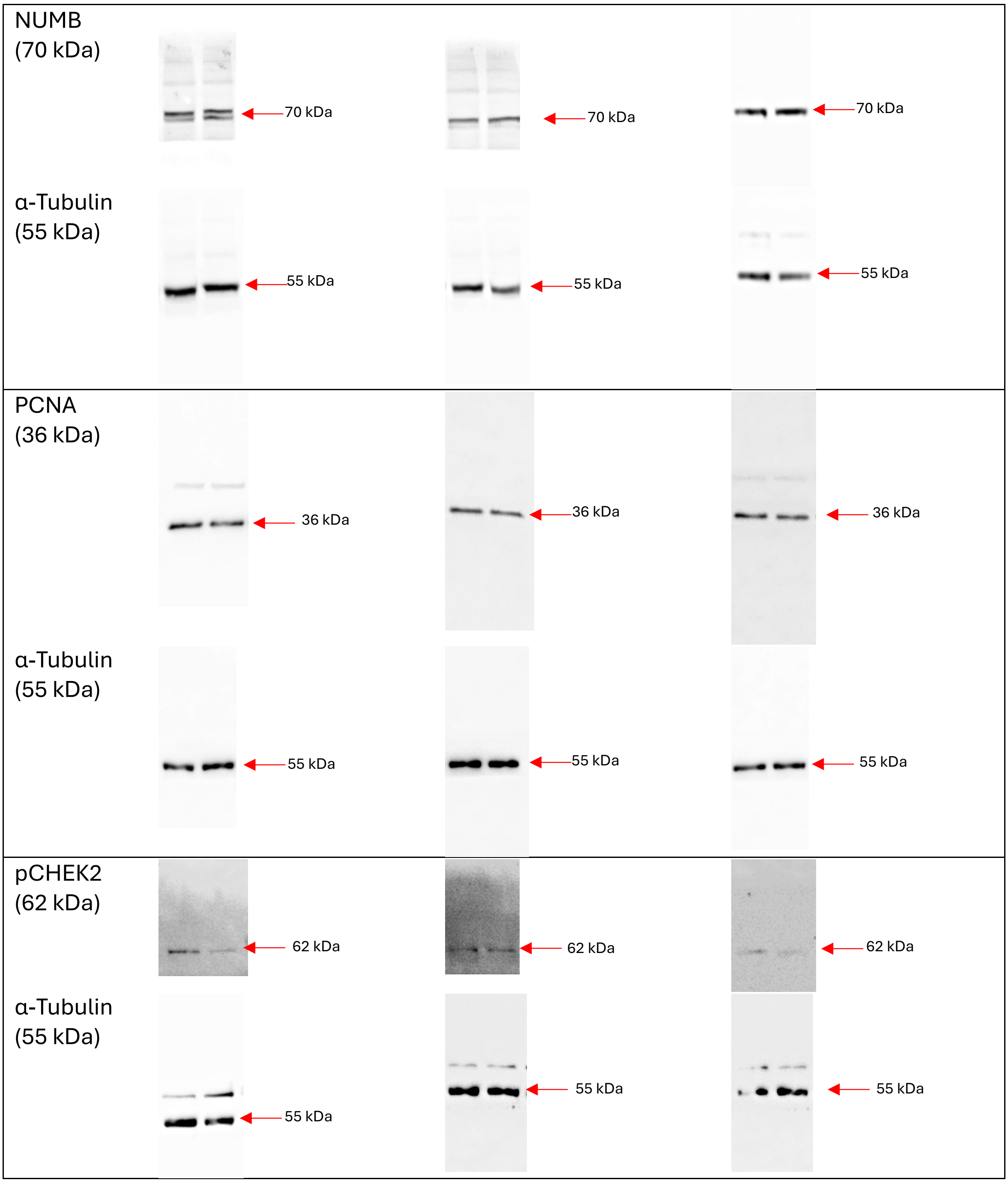
NUMB
(70 kDa)
70 kDa
70 kDa
70 kDa
α-Tubulin
(55 kDa)
55 kDa
55 kDa
55 kDa
PCNA
(36 kDa)
36 kDa
36 kDa
36 kDa
α-Tubulin
(55 kDa)
55 kDa
55 kDa
55 kDa
pCHEK2
(62 kDa)
62 kDa
62 kDa
62 kDa
α-Tubulin
(55 kDa)
55 kDa
55 kDa
55 kDa

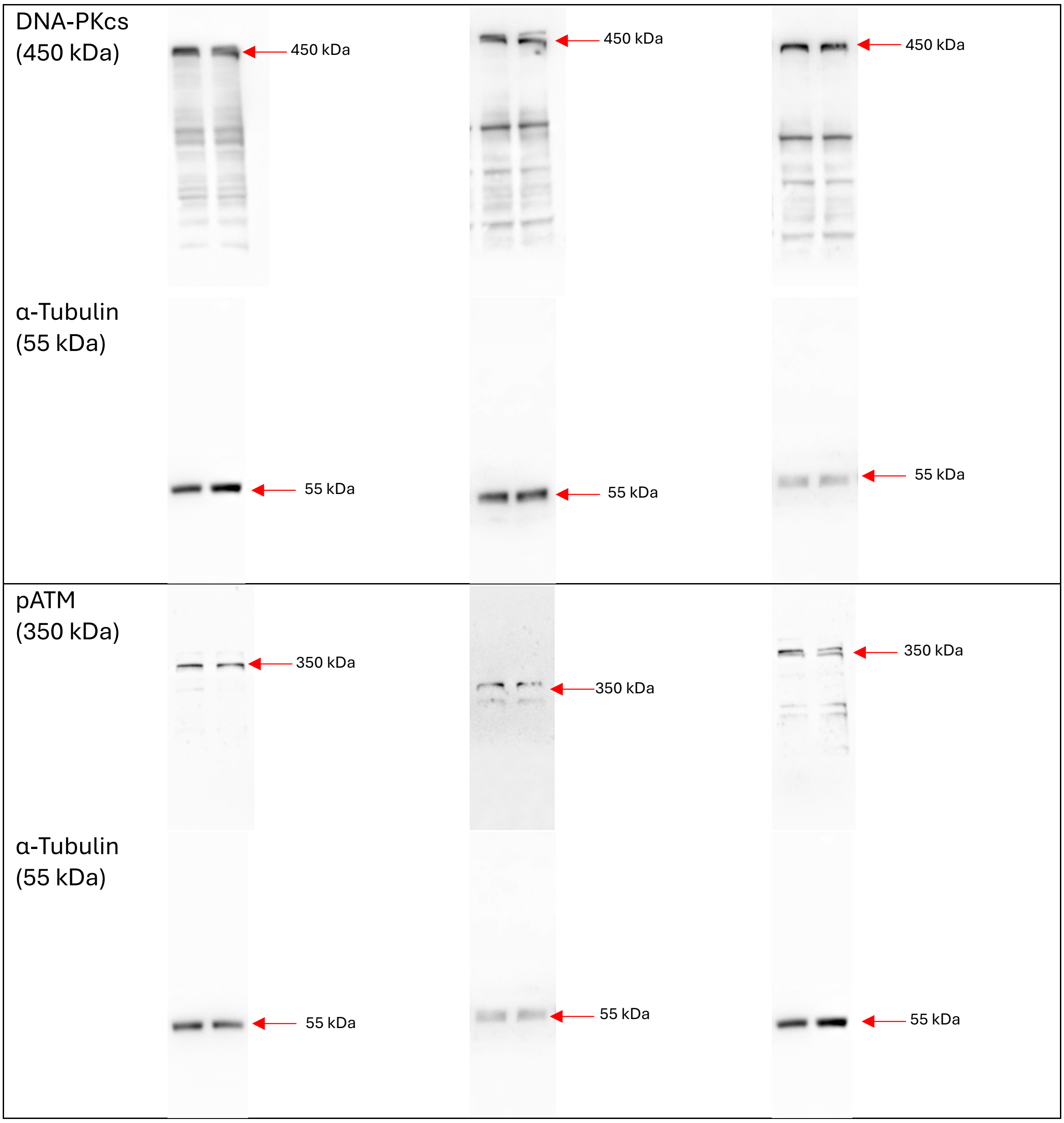
DNA-PKcs
(450 kDa)
450 kDa
450 kDa
450 kDa
α-Tubulin
(55 kDa)
55 kDa
55 kDa
55 kDa
pATM
(350 kDa)
350 kDa
350 kDa
350 kDa
α-Tubulin
(55 kDa)
55 kDa
55 kDa
55 kDa

49

HT	Biological Replicate 1<br>ctrl: left<br>MSI2 KD: right	Biological Replicate 2<br>ctrl: left<br>MSI2 KD: right	Biological Replicate 3<br>ctrl: left<br>MSI2 KD: right
Bcl-xL<br>(30 kDa)<br><br>α-Tubulin<br>(55 kDa)	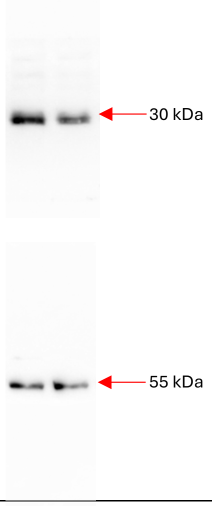	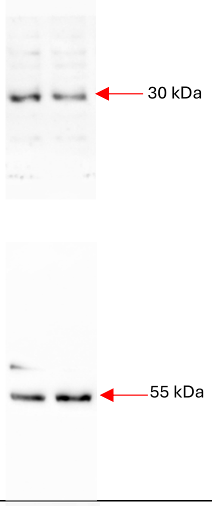	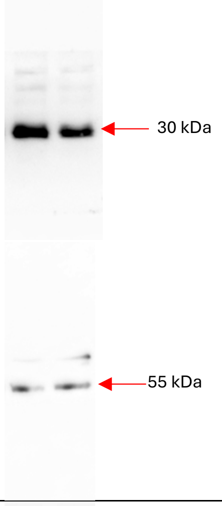
β-Catenin<br>(90 kDa)<br><br>α-Tubulin<br>(55 kDa)	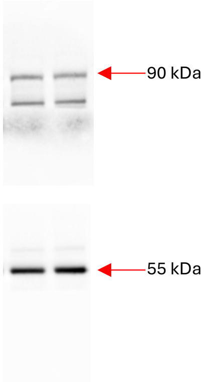	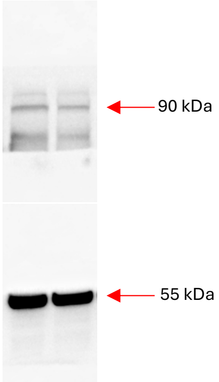	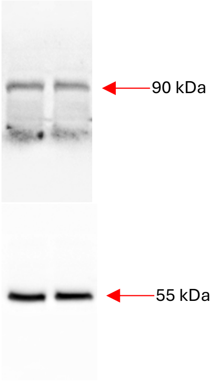
NOTCH-1<br>(120 kDa)<br><br>α-Tubulin<br>(55 kDa)	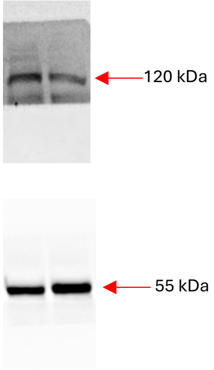	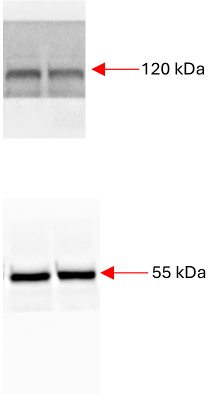	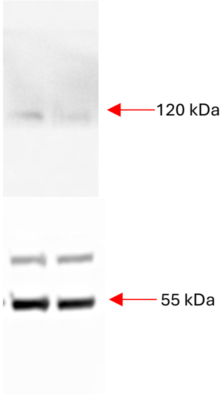

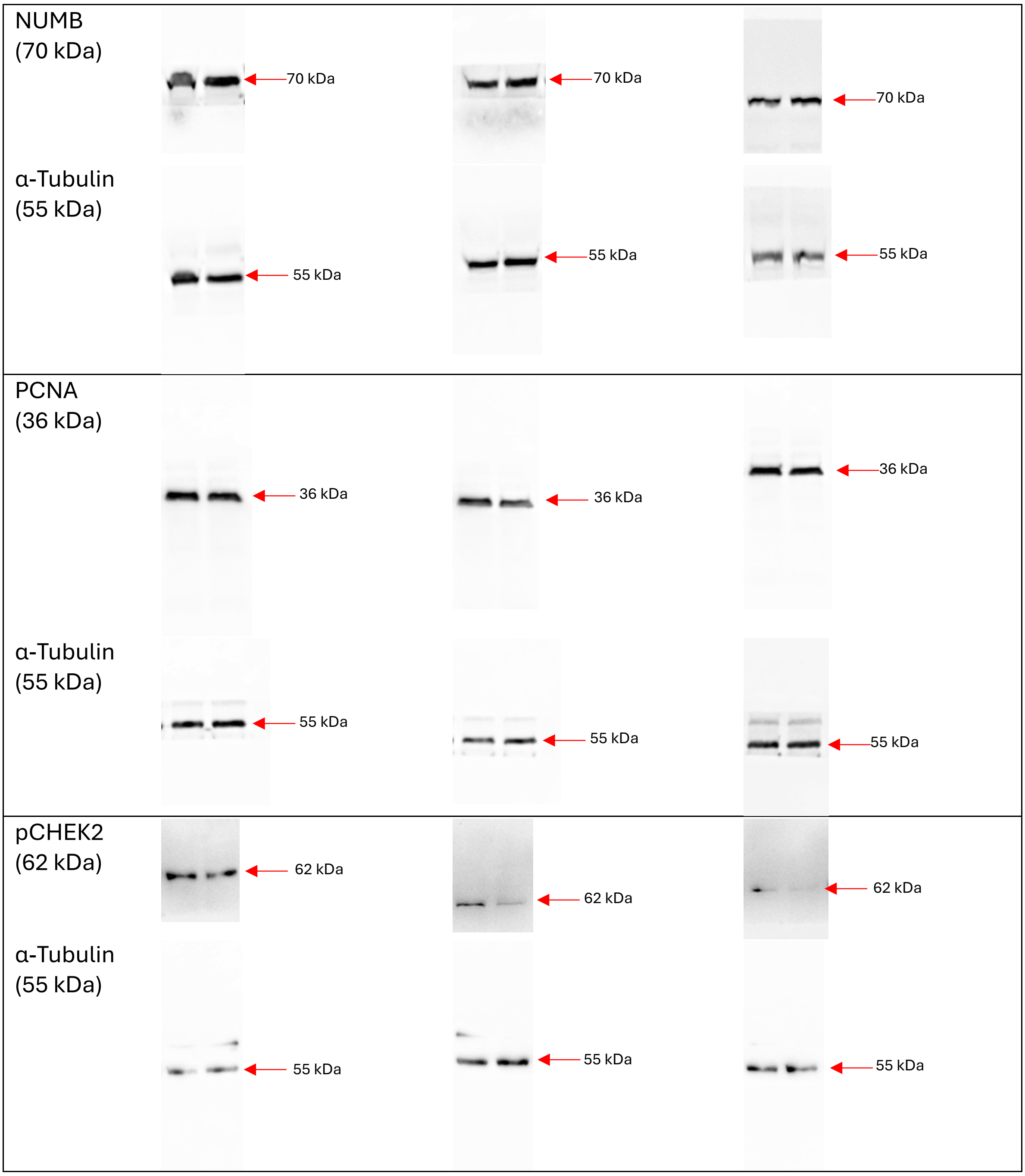
NUMB
(70 kDa)
70 kDa
70 kDa
70 kDa
α-Tubulin
(55 kDa)
55 kDa
55 kDa
55 kDa
PCNA
(36 kDa)
36 kDa
36 kDa
36 kDa
α-Tubulin
(55 kDa)
55 kDa
55 kDa
55 kDa
pCHEK2
(62 kDa)
62 kDa
62 kDa
62 kDa
α-Tubulin
(55 kDa)
55 kDa
55 kDa
55 kDa

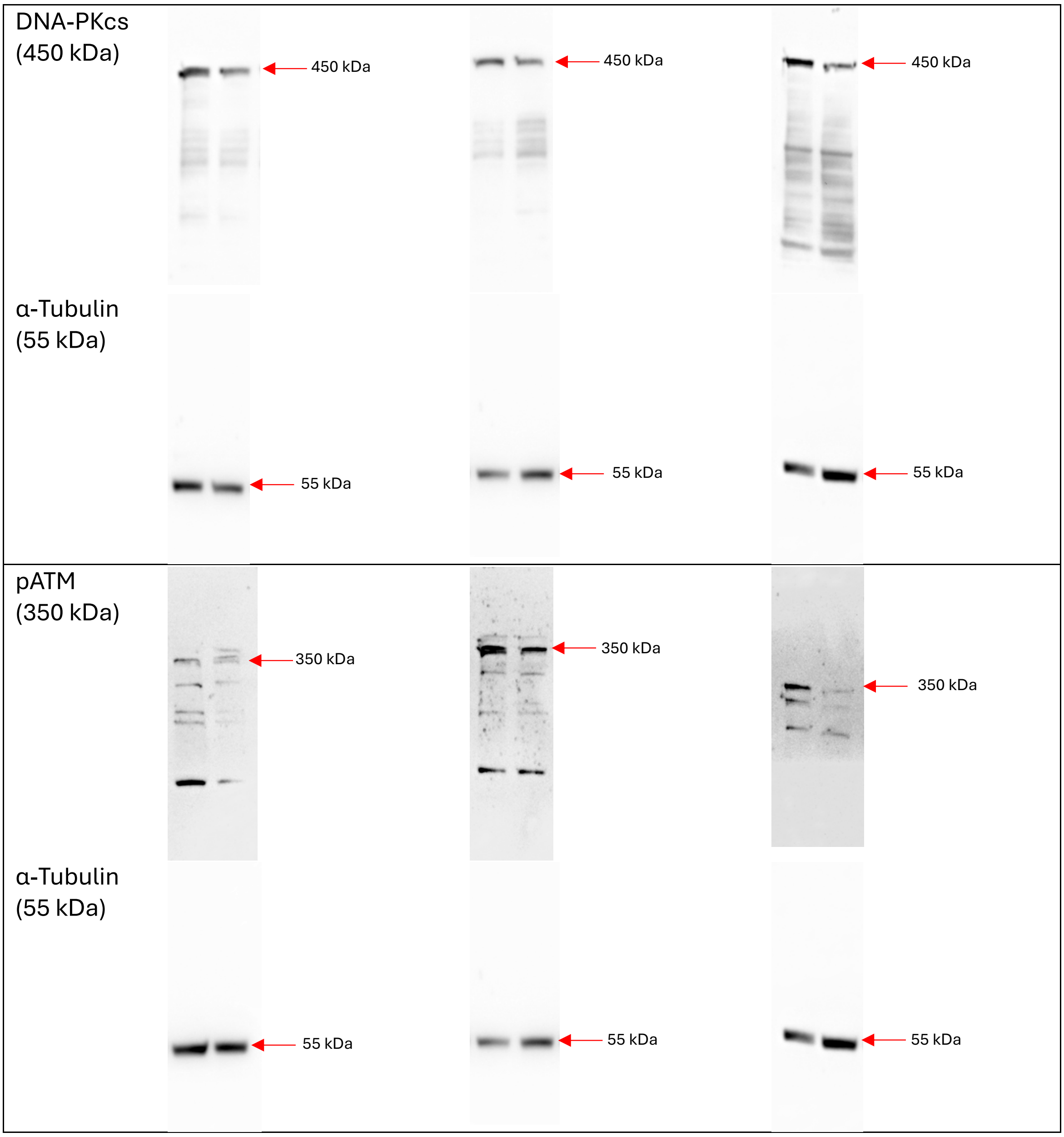

Supplementary Figure 6: Exemplary biological replicates of western blotting experiments. Exemplary blots are shown in biological replicates for all tested proteins in both cell lines. Depicted are blots in control condition (left) and MSI2 KD condition (right) in all images. The corresponding Tubulin blots are depicted below each protein.

Supplementary Figure 7: Biological replicates of DNA damage response phosphorylation array

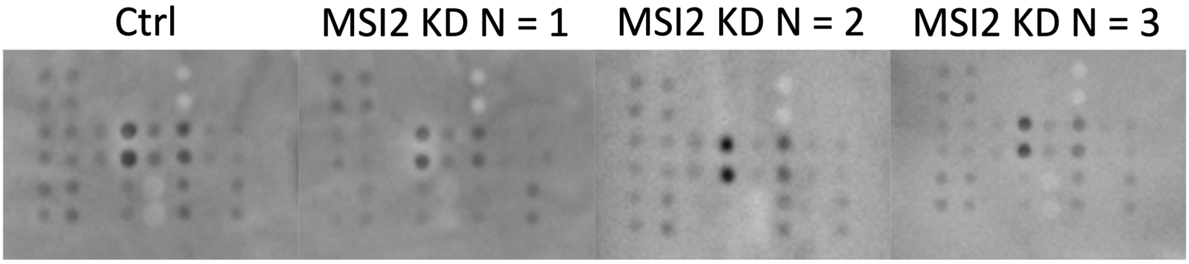

Supplementary Figure 7: Biological replicates of DNA damage response phosphorylation array. DNA damage response phosphorylation array is shown in biological replicates tested in HT cell line. Depicted is the control condition (left) and three biological replicates in MSI2 KD condition (right). The relevant altered proteins and positive/negative controls can be compared to in **Figure 6 D, E & F**, or in the manufacturers' protocol.
