# Supplementary material for: Prognostic marker Musashi-2 modulates DNA damage response and radioresistance in diffuse large B-cell lymphoma
Source: Front Cell Dev Biol. 2025 Aug 6;13:1575483. doi: 10.3389/fcell.2025.1575483 (PMC12364875; doi:10.3389/fcell.2025.1575483)
Supplement: Supplementary file 2 [file DataSheet1.pdf]

Supplementary Methods

Gene expression analyses

To determine Musashi baseline expression, we used gene expression profiling from 114 B-cell NHL patients provided in the GSE32018 dataset (Gómez-Abad et al., 2011). The database contains gene expression data from different NHLs as well as non-malignant tonsil or lymph node samples. Analyses were based on normalized log2 ratio data as provided by the authors. IDs A_23_P139795 (MSI1) and A_23_P369479 (MSI2v2) were used. Comparisons were performed using the Wilcoxon test.

MSI1 and MSI2 gene expression in eight established DLBCL cell lines was analyzed using the GSE50721 dataset by Hardee et al. (Hardee et al., 2013).

For correlation analyses and expression comparisons within a DLBCL cohort, we used the largest available dataset including gene expression profiling from 1310 diagnosed DLBCL patients provided by Care and Barrens, GSE181063 (Painter et al., 2019; Lacy et al., 2020). We used normalized data generated via variance stabilizing transformation, as provided by the authors. For each target, the sequence ID with the highest expression within the dataset was used. Hence, analyses were based on IDs ILMN_1739601 (MSI1), ILMN_1713088 (MSI2 transcript variant 1), and ILMN_1804448 (MSI2 transcript variant 2).

For survival analyses, four datasets were used as described in the main manuscript. Importantly, including the IPI score in the multivariable model for the GSE181063 dataset was not feasible due to (1) high missingness of data > 30%, and (2) non-random missingness, as IPI scores were mostly not available for patients with a short life expectancy (log rank $p < 0.001$ for survival comparisons between patients with and without available IPI score). Intriguingly, MSI2 transcript variant 2 expression was also significantly higher in patients with missing IPI scores than in those with available IPI scores ($p = 0.01$ in Mann Whitney U testing), suggesting that inclusion of IPI scores in our model would have resulted in substantial statistical bias. Hence, IPI score was left out of the multivariable model for the GSE181063 dataset. Otherwise, models containing MSI2 expression and IPI score as well as DLBCL subtyping were consistently used. Notably, only the GSE181063 contained different sequences for MSI2 transcript variants so, for the remaining three datasets, the overall MSI2 sequence with the highest expression was used.

For co-expression gene list generation, we used custom settings as proposed by the UALCAN tool for analysis of the TCGA-DLBC dataset. Specifically, all genes showing positive correlations with a Pearson Correlation Coefficient (PCC) of at least 0.3 and with a median Transcripts Per Million count ≥0.5 were included. For the analysis using the Cancer Hallmark tool, the resulting gene list was compared against a pre-defined set of 1574 genes, the "Core cancer hallmark gene set" and overrepresentation was assessed by the tool.

References

Gómez-Abad, C., Pisonero, H., Blanco-Aparicio, C., Roncador, G., González-Menchén, A., Martinez-Climent, J. A., et al. (2011). PIM2 inhibition as a rational therapeutic approach in B-cell lymphoma. *Blood* 118, 5517–5527. doi: 10.1182/blood-2011-03-344374

Hardee, J., Ouyang, Z., Zhang, Y., Kundaje, A., Lacroute, P., and Snyder, M. (2013). STAT3 Targets Suggest Mechanisms of Aggressive Tumorigenesis in Diffuse Large B-Cell Lymphoma. *G3 Genes|Genomes|Genetics* 3, 2173–2185. doi: 10.1534/g3.113.007674

Lacy, S. E., Barrans, S. L., Beer, P. A., Painter, D., Smith, A. G., Roman, E., et al. (2020). Targeted sequencing in DLBCL, molecular subtypes, and outcomes: a Haematological Malignancy Research Network report. *Blood* 135, 1759–1771. doi: 10.1182/blood.2019003535

Painter, D., Barrans, S., Lacy, S., Smith, A., Crouch, S., Westhead, D., et al. (2019). Cell-of-origin in diffuse large B-cell lymphoma: findings from the UK's population-based Haematological Malignancy Research Network. *Br J Haematol* 185, 781–784. doi: 10.1111/bjh.15619
